# Discovery of a new species of Adder’s tongue fern from India with comparative analysis of morphological and molecular attributes

**DOI:** 10.1038/s41598-021-03231-w

**Published:** 2021-12-22

**Authors:** B. L. Yadav, Mukesh K. Meghvansi, Kanta Meena, C. B. Gena

**Affiliations:** 1 Department of Life Science, Mewar University, Gangrar, Chittorgarh, Rajasthan 312901 India; 2 Bioprocess Technology Division, Defence Research and Development Establishment, Gwalior, Madhya Pradesh 474002 India; 3 Department of Botany, Manikya Lal Verma Government College, Bhilwara, Rajasthan 311001 India; 4 Maharaja Ganga Singh University, Bikaner, Rajasthan 334004 India

**Keywords:** Plant sciences, Natural variation in plants, Taxonomy

## Abstract

Eusporangiate fern genus *Ophioglossum* L. is commonly known as Adder’s tongue fern as its fertile frond gives the appearance of snake tongue. A new species in this fern genus, *O. trilokinathii* belonging to Ophioglossaceae family has been discovered from the plateau region of Rajasthan State of northwestern India. The new species can be distinguished from other taxa of this genus by its smaller habit, subglobose-tuberous rhizome, basipetal emergence of young roots, aggregation of old decaying roots on rhizome apex, fertile stalk as well as spike short and thick, trophophylls in rosette, ovate or orbicular and a unique sporoderm sculpture pattern under SEM having broad reticulations with thick and raised muri enclosing large hexagonal or irregular areas on the distal and proximal faces of the spores hitherto unreported in any of the presently known taxa of *Ophioglossum*. In addition, comparative study of stomatal structure, foliar anatomy and nucleotide sequence data of its three chloroplast DNA markers (*trnL-F*, *rbcL* and *psbA-trnH*) was carried out. In view of all the attributes including habitat, ecology, morphology, foliar anatomy, stomatal features, palynology and molecular phylogenetic data, the present study suggests that the *Ophioglossum* specimen collected from plateau region of Rajasthan represents a hitherto undescribed species thereby warranting its establishment as *O. trilokinathii* sp. nov. A detailed comparative account of the new taxon with its allied species has also been provided.

## Introduction

The fern genus *Ophioglossum* L. is cosmopolitan in distribution and is peculiar in having certain non-fern characters like absence of circinate vernation in leaf, lack of sclerechyma in the entire plant body, presence of special reproductive organ—spike and presence of petiolar collateral and cauline vascular bundles^1^. Furthermore, the occurrence of usually large chromosome numbers in *Ophioglossum* has made it more popular among the researchers studying the plant biological systems. The report of n = 720 (2n = 1440) in a population of *O. reticulatum* L. from Shevaroy Hills, south India^2^ is the highest chromosome number possessed by any plant or animal species in the biological world. *Ophioglossum* is a very challenging taxon with regard to species delimitation and identification due to its simple plant body represented by underground rhizome (except in the epiphytic species *O. pendulum and O. palmatum*) and an aerial complex which consists of sterile and fertile fronds. The classification of its species is based on such features of the sporophyte as leaf size, shape, venation, features of spike and spores which are not ordinarily suffice for systematic purposes in other groups^3^. Whereas, the classification is primarily based on the leaf attributes, the other parts of the sporophyte being buried under the ground thus, not available for systematic purposes can potentially lead to a greater taxonomic ambiguity. Furthermore, morphological plasticity is well known in species of this genus. Sometimes the intraspecific variations due to variable microclimatic conditions of the habitat or polyploidy, are so prominent that they appear to be a distinct species^4^. This is evident from the total number of species which greatly varies from 26 to 54 under this genus^5–9^. Presently, the genus is represented by 52 species world over^10^, of which more than 20 are reported from different bio-geographic regions of India^11–23^.

Rajasthan, the largest State lying in the northwestern part of India has four major physiographic regions namely, the Western Thar Desert, the Aravalli Range with Vindhyan Mountains, the Eastern plains and Southeastern plateau region. The southeastern plateau region locally called as *Pathar* or *Upar Mall* region is a humid zone with rich floristic diversity^24–26^. Mainal area of Chittorgarh district is the part of this plateau region and is home of several lycophytes and ferns too^27,28^. Of the eight *Ophioglossum* species (*O. costatum* R. Br., *O. gramineum* Willd., *O. gujaratense* Patil, Kachhiyapatel, Patel & Rajput, *O. indicum* Yadav & Goswami, *O. lusitanicum* L., *O. parvifolium* Grev. & Hook., *O. petiolatum* Hook. and *O. reticulatum* L.) reported from this state^29–32^, six have been found to occur in the Mainal area^31,32^. Thus, this area happens to be a potential nano hot spot with regard to Adder’s tongue fern diversity. However, limited efforts have been made on extensive survey and detailed investigation for generating the comprehensive data pertaining to species diversity, morphology, palynology, anatomy and molecular biology of this interesting fern genus *Ophioglossum*. In a botanical excursion carried out during 2016–2018 with an aim to study species diversity and the field behaviour of various taxa of this genus, authors came across an interesting population of *Ophioglossum* in Mainal area. Initially, the plant specimens of this population were looking superficially similar to *O. parvifolium* and *O. gujaratense*. However, after comparing the morphological, palynological, anatomical and molecular characters (*trnL-F*, *rbcL* and *psbA-trnH* chloroplast DNA markers) of collected plants with other closely allied species of *Ophioglossum,* it is concluded that the population of Mainal stands as an undescribed taxon. Hence the new taxon is described as *O. trilokinathii* sp. nov.

## Results

### Taxonomy

**Figure 1:**
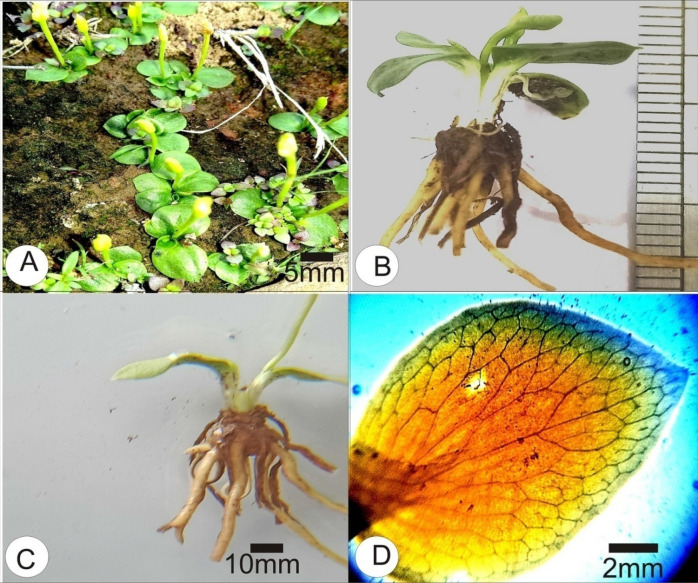
*Ophioglossum trilokinathii* sp. nov. **(A)** Habitat. **(B)** Entire plant with aggregation of roots of previous season (older roots) at rhizome apex. **(C)** Rhizome showing basipetal emergence of roots. **(D)** Trophophyll-size, shape and venation.

**Figure 2:**
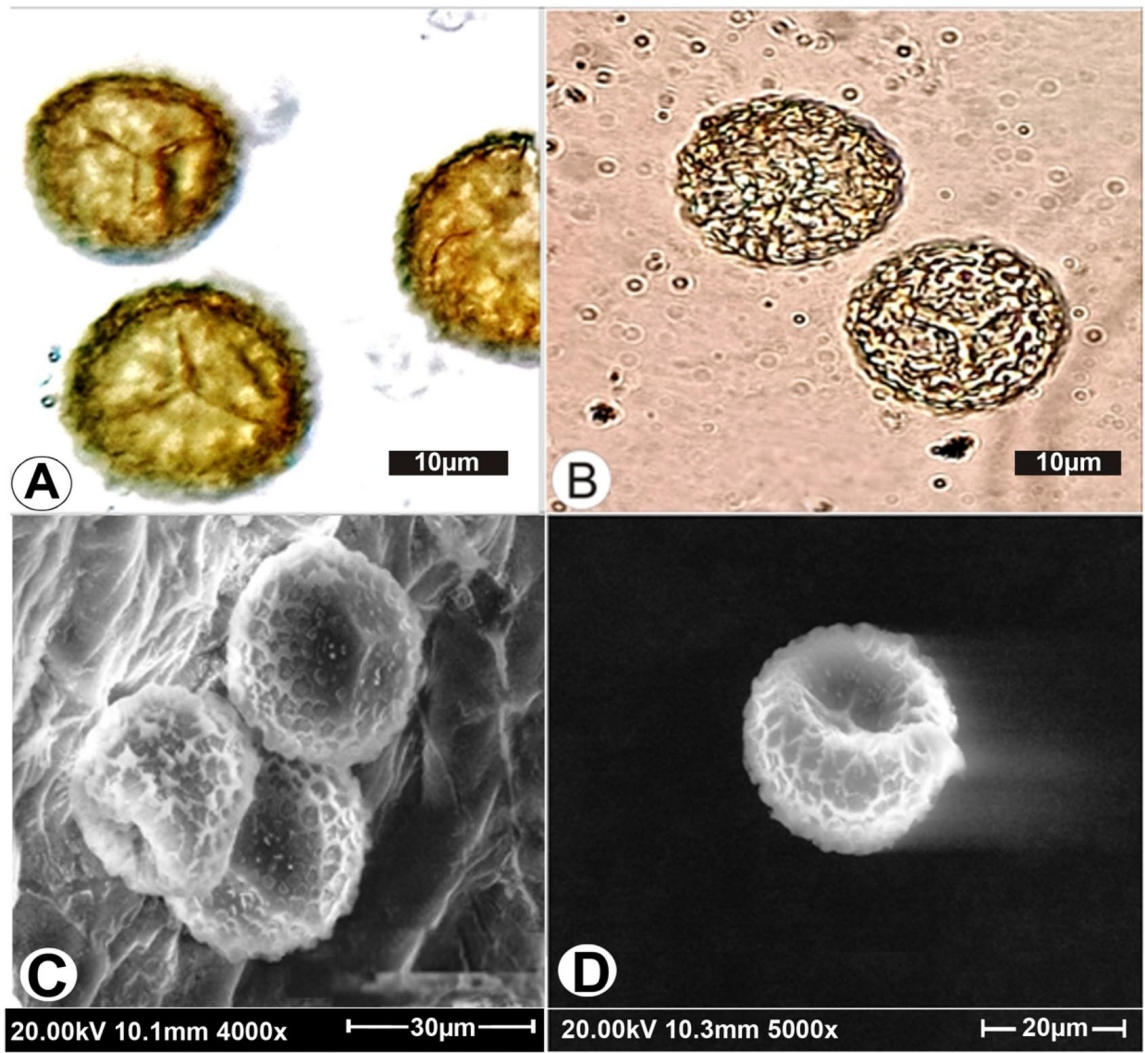
Spore structure of *Ophioglossum trilokinathii* sp. nov. **(A)** Spores with distinct layers of exine and perine. **(B)** Spores with distal and proximal face with triradiate mark. **(C)** SEM image of spores—exine ornamentation. **(D)** SEM image of spore—triradiate mark in the central cavity of proximal face.

*Ophioglossum trilokinathii*. B L Yadav, M K Meghvansi, K Meena & C B Gena sp. nov. (Fig. 1 and 2).

#### Type

INDIA. Rajasthan: Chittorgarh district, Mainal 25° 5^′^ 15^′′^ N, 75° 10^′^ 01^′′^ E, elevation ~ 507 m, 12 August, 2016, *B. L. Yadav, C. B. Gena & Kanta Meena* 65.

#### Holotype

Central National Herbarium, Kolkata (CAL!).

#### Isotype

Herbarium Botanical Survey of India, Arid Zone Regional Centre, Jodhpur (BSJO!); Herbarium Mewar University, Chittorgarh, Rajasthan (MUCR 0036).

#### Type locality

Mainal (25° 5^′^ 15^′′^ N, 75° 10^′^ 01^′′^ E, elevation ~ 507 m) Chittorgarh district, Rajasthan, northwestern India.

#### Etymology

The specific epithet has been chosen in honour of Professor Triloki Nath Bhardwaja (Professor T. N. Bhardwaja), Former Vice-Chancellor, V. M. Open University, Kota, Rajasthan, India for his commendable work in the field of pteridology.

#### Diagnosis

*O. trilokinathii* sp. nov. is unique among the species of this genus in having basipetal emergence of roots, aggregation of old decaying roots at rhizome apex, trophophylls in rosette touching the substratum, peculiar broad reticulate spore wall ornamentation with thick and raised muri enclosing large hexagonal or irregular areas, granulose perine (perispore), differentiation of mesophyll cells into palisade and spongy tissues and elliptic stomata with thin and smooth inner margin of outer stomatal ledge.

#### Description

Plants terrestrial, small, 1.1–2.4 cm in height (Fig. 1A); rhizome subglobose-tuberous, 0.3–0.5 cm long, 0.1–0.2 cm broad (Fig. 1B); roots thick, fleshy, brownish white, stoloniferous, young roots on basal part of rhizome, aggregation of old decaying roots on rhizome apex (Fig. 1B,C); common stalk 0.2–0.4 cm, subterranean, white (Fig. 1B); trophophylls1–4 sometimes 5, arranged in a rosette, horizontal touching the substratum, broadly ovate or ovate orbicular, thick, margin entire, apex acute or apiculate, base cuneate, lamina surface sometimes with 1–3 longitudinal shallow furrows, midrib absent, 0.6–0.9 cm long, 0.4–0.7 cm broad (Fig. 1D); venation reticulate, marginal aeroles with free vein endings (Fig. 1D); fertile segment arises from the junction of face of lamina and common stalk, 0.7–1.5 cm long, thick, spike short, thick, 0.4–0.9 cm long, with two rows of lateral sporangia and a sterile tip, sometimes sterile tip absent; sporangia 5–10 pairs; spores trilete, globose, perinnate (Fig. 2A), 18–30 µm in size, laesural arms short, straight not reaching to the margins of central cavity of proximal face, sporoderm reticulate under Light Microscope (Fig. 2A,B).

SEM imaging revealed that spores were globose and perinate. Interestingly, spores have a unique exine ornamentation pattern of broad reticulations with thick and raised muri, enclosing large hexagonal or irregular areas on distal and proximal faces, proximal face with distinct short tri-radiate mark and reticulations, laesural arms smooth and straight, not reaching to the margins of cavity of proximal face. To the best of our knowledge, the exine ornamentation pattern as noted in this case has not been reported so far in any other presently known species of *Ophioglossum* (Fig. 2C,D).

#### Reproductive period

July–August.

#### Distribution and ecology

India—Rajasthan state, Chittorgarh district, Mainal locality. The species grow in dense populations on moist soil in open terrestrial habitat at an elevation of ~ 507 m. Plants of this species sprout from the underground rhizome after a fortnight period of first showers in the month of June– July and dry up by the mid of September every year.

#### Conservation status

The species has been recorded from Mainal area which is known for its famous waterfall and nearby temple of Jogniyamata. It is a plateau region supporting the occurrence of *O. costatum*, *O. gramineum*, *O. parvifolium*, *O. indicum*, *O. petiolatum*, and *O. gujaratense.,* Therefore it is one of the richest localities of Adder’s tongue fern where contiguous occurrence of two or more species is frequent. The species population occurs in 1.0 × 1.0 m^2^ area and is represented by 300–350 individuals. Future explorations are needed to get its entire range of distribution and therefore, at present the species is treated under the category “Data Deficient” (DD) of IUCN^33^.

### Species recognition

Comparison of the new taxon with its allied species provided in Table 1 reveals that *O. trilokinathii* sp. nov. partially resembles morphologically with *O. hitkishorei* Patel & Reddy in such features as thick trophophyll, acute apex and cuneate base. However, *O. hitkishorei* differs from *O. trilokinathii* sp. nov. by its marshy habitat, larger size, roots non stoloniferous, acropetal emergence of young roots, trophophylls upward from the ground, spore exine shows beaded strings of exine grains forming polygonal or round spaces on the distal pole and granulate proximal face^23^.

**Table 1:** Comparison of morphological characters among *O. trilokinathii sp.nov*., *O. hitkishorei, O. costatum O. gujaratense* and *O. parvifolium.*

S. no.	Characters	*O. trilokinathii* sp. nov.	*O. hitkishorei*	*O. costatum*	*O. gujaratense*	*O. parvifolium*
1.	Habitat	Terrestrial	Marshy	Terrestrial	Terrestrial	Terrestrial
2.	Plant height (cm)	1.1–2.4	3.0–8.0	8–10	Less than 10.0	Up-to 10.0
3.	Rhizome shape	Subglobose-tuberous	Globose–subglobose	Subglobose with apical cupule	Tuberous	Subglobose-tuberous
4.	Root	Basipetal emergence, stoloniferous	Acropetal emergence, non stoloniferous	Basipetal emergence, non stoloniferous	Acropetal emergence, stoloniferous	Acropetal emergence, stoloniferous
5.	**Trophophyll**					
	Number	1–4	2–6	1–6	1–4	1–2
	Shape	Ovate or orbicular	Elliptic, ovate oblanceolate	Elliptic- lanceolate	Ovate elliptic, elliptic-lanceolate	Ovate, elliptic, sometimes orbicular
	Position	Flat and touching to soil surface	Upward from the ground	Upward from the ground	Horizontal slightly above ground	Flat on substratum
6.	Fertile stalk Length (cm)	0.7–1.7	2.5–5.8	4.3–5.8	2.0–8.0	1.8–3.2
7.	Spore size	18–30	20—30	36–38	19–28	31–35
8.	Exine ornamentation	Muri thick, form large hexagonal on distal and proximal face, proximal face not granulate, laesural arms straight, short not reaching to the margins of central cavity	Beaded strings of exine grains form hexagonal areas on distal face, Proximal face granulate, laesural arms straight up to margins of the central cavity	Exine reticulated on distal face and porate on proximal face, muri vermiculate enclosing deep polygonal lumina, surface of muri rough. Laesural arms upto the margins of central cavity	Reticulated muri of uneven heights enclosing shallow areas on distal face and distinct reticulation on proxoimal face, laesural arms prominent sometimes wavy reaching to the margins of central cavity	Muri wide enclosing shallow conical lumina on distal face, pitted ornamentation on proximal face. Laesural arms straight upto the margins of central cavity

**Figure 3:**
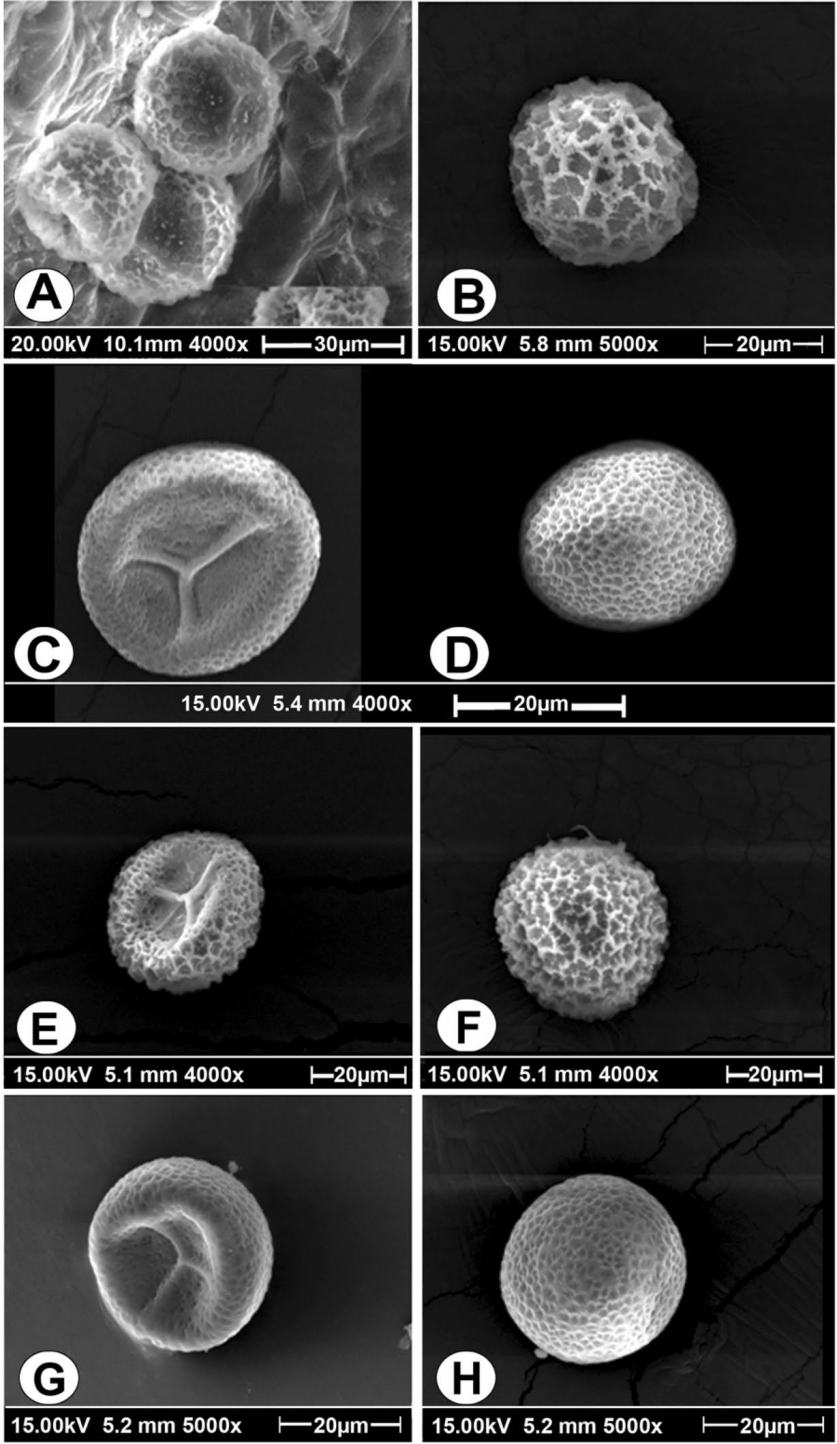
Comparison of spore structure of some species of *Ophioglossum* under SEM. **(A)** *O. trilokinathii* sp. nov. proximal face. **(B)** *O. trilokinathii* sp. nov. distal face. **(C)** *O. costatum* proximal face. **(D)** *O. costatum* distal face. **(E)** *O. gujaratense* proximal face. **(F)** *O. gujaratense* distal face. **(G)** *O. parvifolium* proximal face. **(H)** *O. parvifolium* distal face.

Basipetal emergence of young roots and 1–4 or 5 trophophylls are also found in *O. costatum* but in this species bottom part of subglobose rhizome is devoid of roots, roots non stoloniferous, trophophylls are larger with distinct midrib, not arranged in rosette form and reticulate exine with vermiculate muri, muri thin and raised enclosing deep polygonal lumina on distal face and almost pitted ornamentation on proximal face. Leasural arms of triradiate ridge straight reaching to the margins of the central cavity of spore (Fig. 3C,D) which has also been described by earlier workers^34^ whereas exine ornamentation of spores of *O. trilokinathii* sp. nov. is altogether different in having thick and raised muri enclosing large hexagonal or irregular areas on distal and proximal faces, proximal face with distinct short tri-radiate mark and reticulations, laesural arms smooth and straight, not reaching to the margins of cavity of proximal face (Fig. 3A,B).

Comparison of the new species (*O. trilokinathii﻿* sp. nov.) with *O. gujaratense* indicates that these two taxa resemble in having stoloniferous roots, subglobose tuberous rhizome, number of trophophylls which are 1–4 sometimes 5, appressed to the ground and common stalk subterranean but *O. gujaratense* differs from *O. trilokinathii* by its larger size, acropetal emergence of roots, trophophylls lanceolate, fertile stalk thin and long^20,32^ and exine with muri of uneven heights along with some flat and wider areas, enclosing shallow depressions on distal face, proximal face reticulated bearing prominent triradiate ridge, laesural arms straight sometimes wavy, reaching to the margins of the central cavity of the spore (Fig. 3E,F).

*O. trilokinathii.* also has some similarities with *O. parvifolium,* as in these two species roots are stoloniferous, common stalk subterranean, trophophylls horizontal touching the soil surface, trophophyll apex sometimes apiculate but the later species differs from the new taxon in, size (upto 10 cm vs 1.1–2.4 cm), emergence of roots (acropetal vs basipetal) number of trophophylls (1–2 vs 1–4), trophophyll base (cordate vs cuneate), fertile stalk (thin and long vs thick and short). Sporoderm structure of *O. parvifolium* is entirely different from that of *O. trilokinathii.* In former species exine is reticulate, muri thin enclosing funnel shaped lumina on the distal face and on proximal face exine is pitted, laseural arms of triradiate ridge straight reaching to the margins of central cavity of the spore (Fig. 3G,H).

Another noteworthy feature of the new taxon is the mesophyll tissue of its trophophyll which shows differentiation into palisade and spongy parenchyma (Fig. S1A). Mesophyll cells towards upper epidermis are elongate and closely packed while those towards lower epidermis are rounded or ovoid and are loosely arranged with intercellular spaces. Such differentiation is not found in other species (Fig.S1B,C) except *O. nudicaule*.

**Figure 4:**
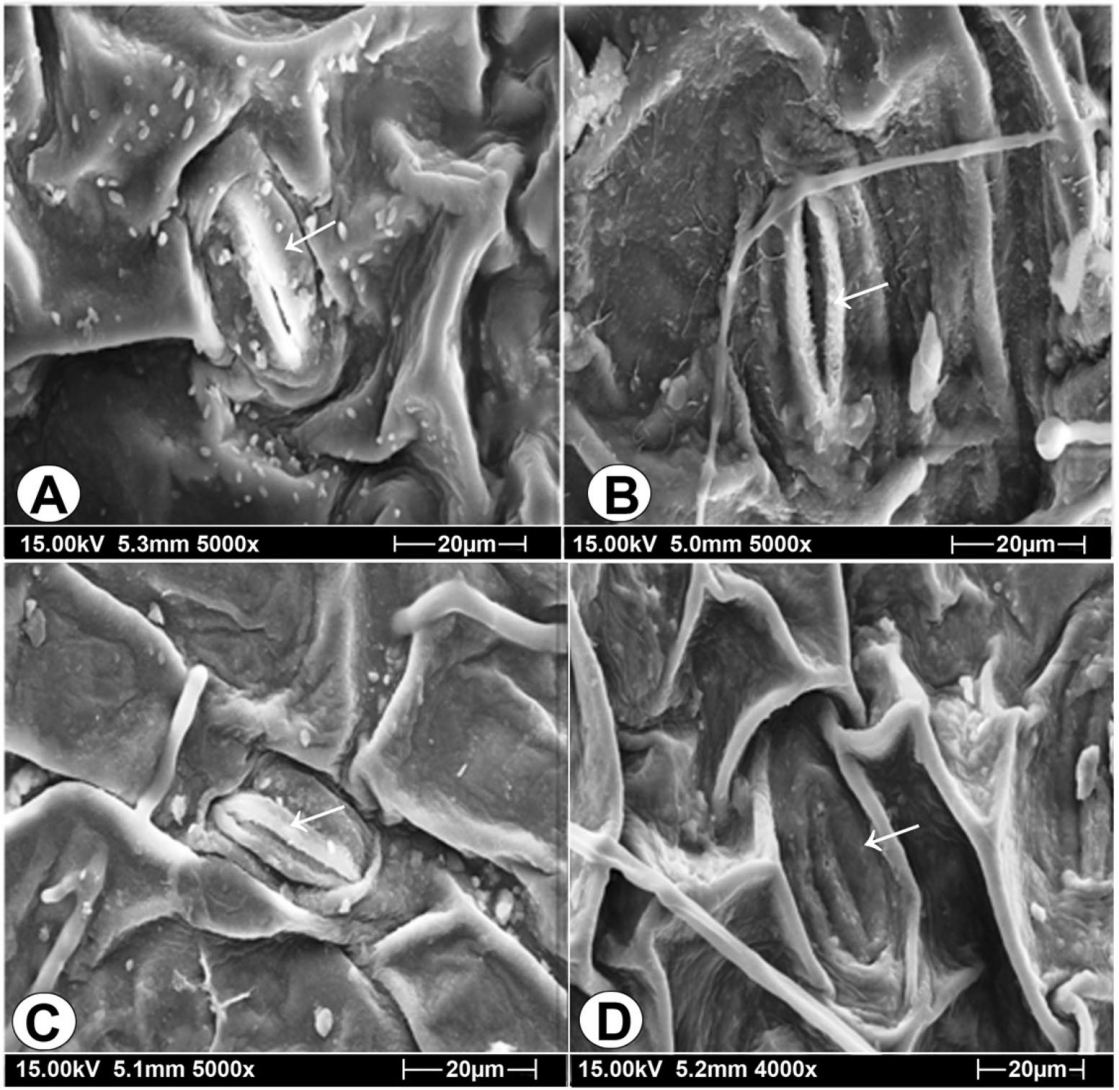
Stomatal features of* Ophioglossum trilokinathii* sp. nov. and its allied species. **(A)** *O. trilokinathii* elliptic stomata with thin and smooth inner margin of outer stomatal ledge. **(B)** *O. costatum* elongated elliptic or lanceolate stomata with thick and hairy inner margin of outer stomatal ledge. **(C)** *O. gujaratense* broad elliptic stomata with thick and rough inner margin of outer stomatal ledge. **(D)** *O. parvifolium* elliptic stomata with thin and striate inner margin of outer stomatal ledge.

The new taxon is quite different from its allied species in its stomatal shape and inner margin of outer stomatal ledge. Trophophylls are amphistomatic. Stomata are elliptic in the new taxon (Fig. 4A) in contrast to the elongated elliptic or lanceolate in *O. costatum*, broadly elliptic in *O. gujaratense* and elongated elliptic in *O. parvifolium*. Inner margin of outer stomatal ledge is thin and smooth in the new species (Fig. 4A) while thick with small hairy structures in *O. costatum* (Fig. 4B), thick and rough in *O gujaratense* (Fig. 4C) and thin and striate in *O. parvifolium* (Fig. 4D).

### Phylogenetic relationship and genetic divergence

**Figure 5:**
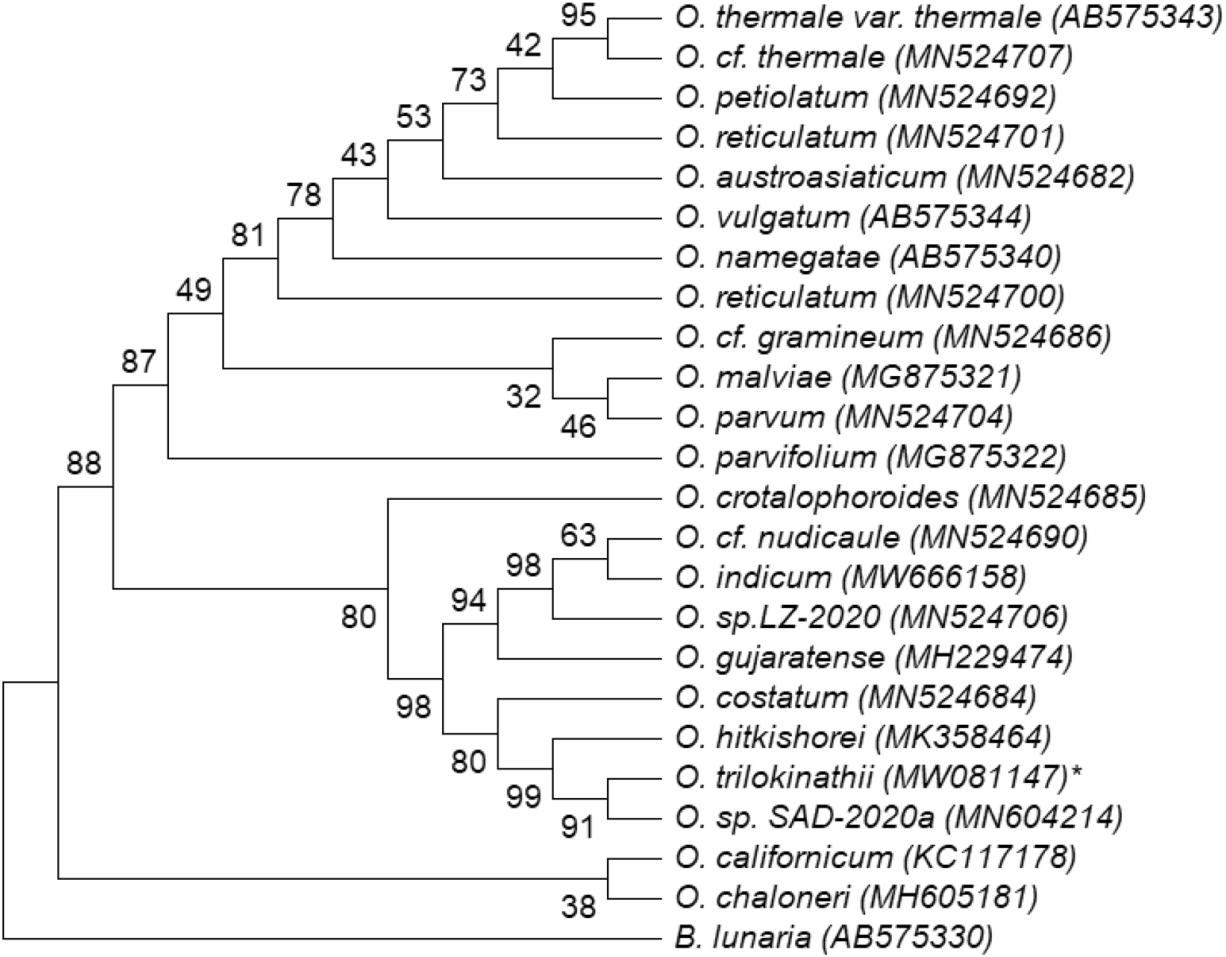
Phylogenetic tree of *Ophioglossum* species based on *psbA-trnH* datasets as analysed using maximum likelihood method. ML bootstrap percentages (BP) are shown on branches.

**Figure 6:**
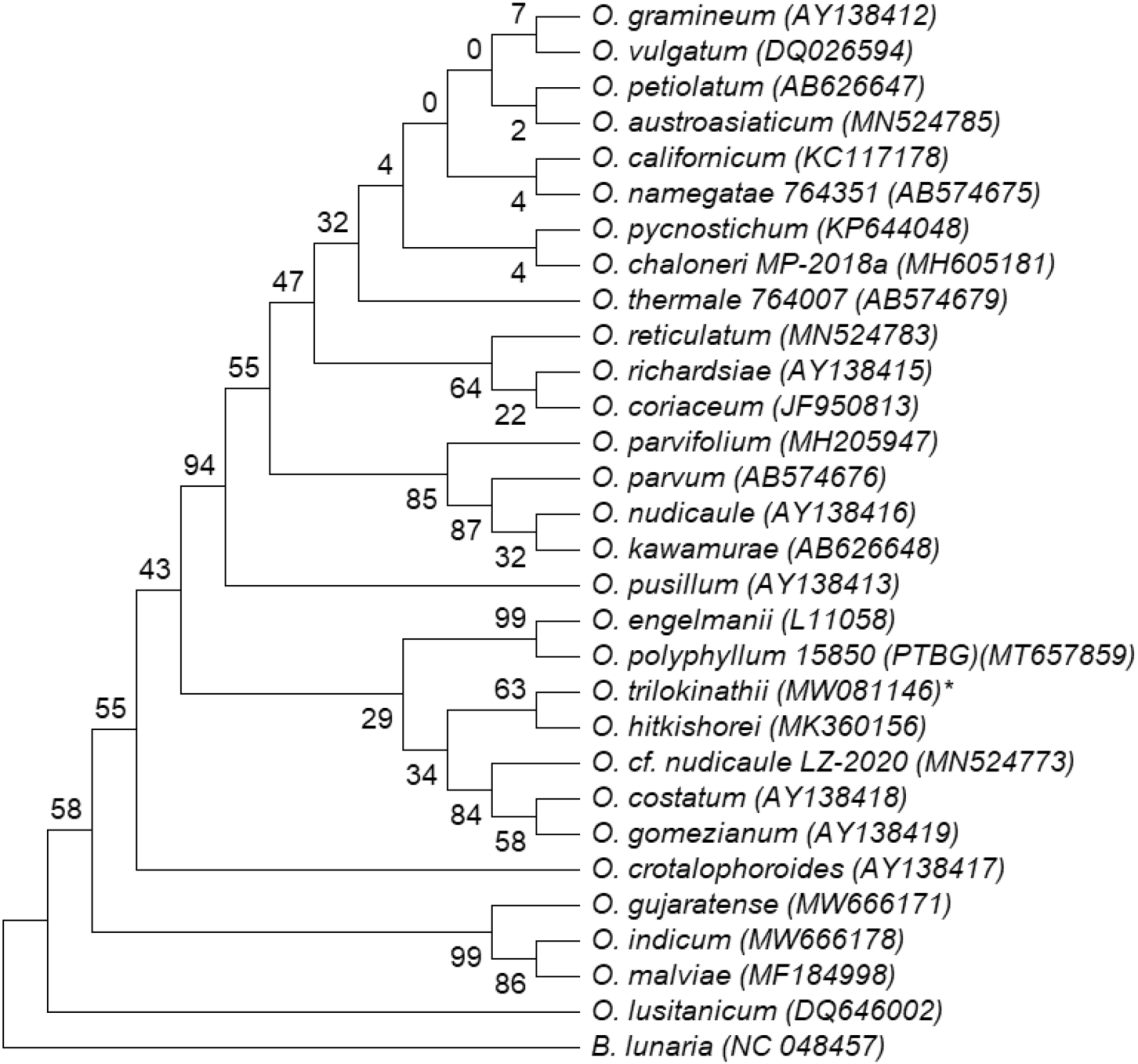
Phylogenetic tree of *Ophioglossum* species based on *rbcL* datasets as analysed using maximum likelihood method. ML bootstrap percentages (BP) are shown on branches.

**Figure 7:**
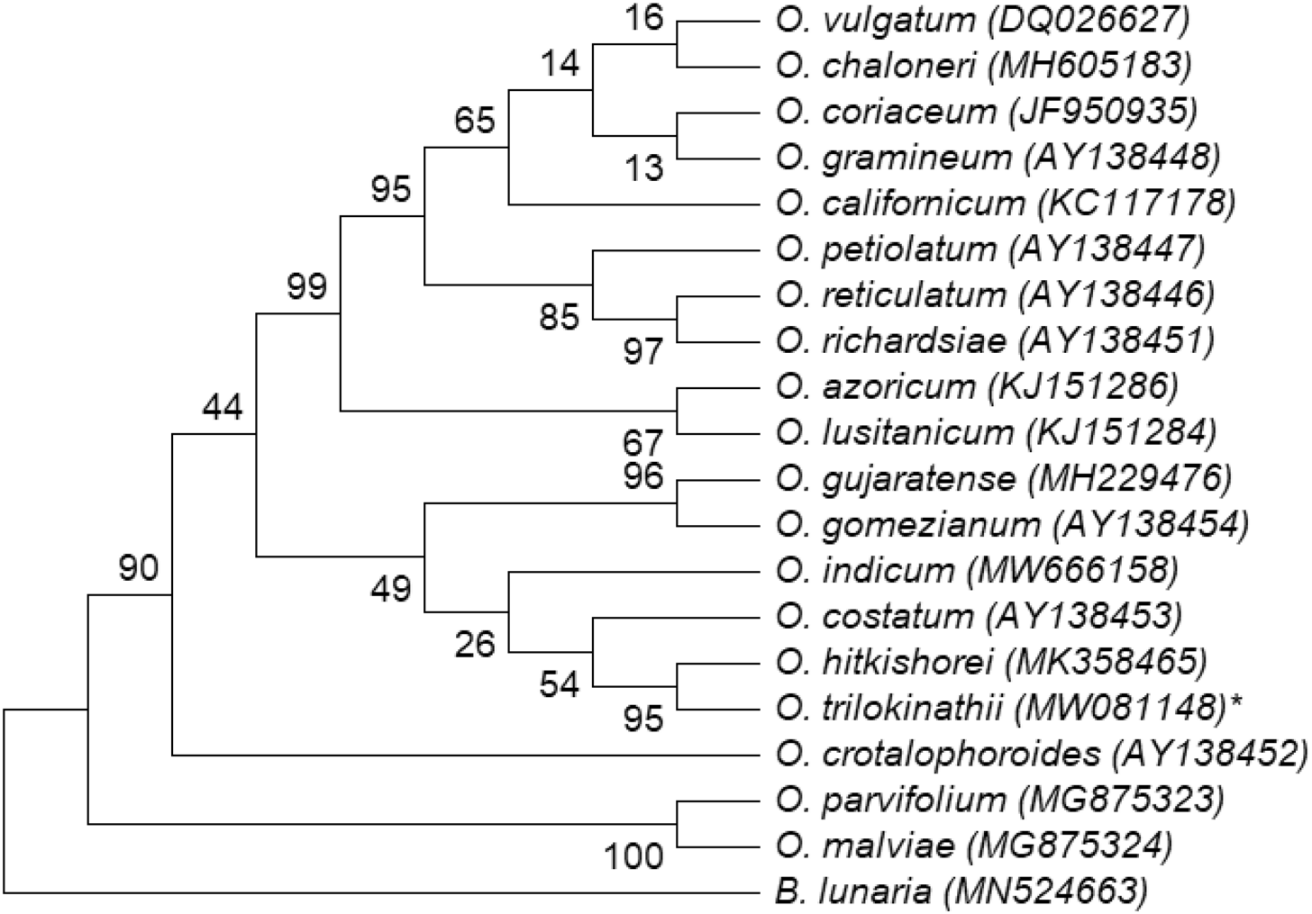
Phylogenetic tree of *Ophioglossum* species based on *trnL-F* datasets as analysed using maximum likelihood method. ML bootstrap percentages (BP) are shown on branches.

Comparison of *psbA-trnH*, *rbcL* and *trnL-F*, nucleotide sequence datasets yielded 91% (Fig. 5), 63% (Fig. 6) and 95% (Fig. 7) ML bootstrap values respectively for *O. trilokinathii* with its closest matching specimens namely *O. sp. SAD-2020a* and *O. hitkishorei*. In addition, the values of evolutionary divergence (p-distance) between *O. trilokinathii* and *O. hitkishorei* with respect to three markers were 0.010 (*psbA-trnH;* Supplementary Table S1), 0.044 (*rbcL;* Supplementary Table S2), and 0.00 (*trnL-F;* Supplementary Table S3) as calculated using MEGA X. Further, the determination of percent identity matrix of nucleotide sequences belonging to *rbcL* and *trnL-F* regions revealed that *O. trilokinathii* exhibited 93.10% and 97.22% identity with *O. hitkishorei* respectively (Supplementary Table S4 & S5). With regard to *psbA-trnH* region, *O. trilokinathii* exhibited 97.15% and 98.86% identity with *O. hitkishorei* and *O. sp. SAD-2020a* respectively (Supplementary Table S6).

## Discussion

*Ophioglossum* is a fern well known for confused state of affairs prevailing in respect of inter-specific differences^5^. The taxonomy of this genus is based on such morphological characters as size of plants, shape and number of leaves, size of spike, number of sporangia per spike which are easily amenable to climatic factors and pose practical difficulties in species delimitation. Thus, besides morphological characters of the plant, palynological, molecular and micromorphological features may be of considerable help in solving the taxonomic tangles in this genus. A comparison of morphological features of the new taxon with *O. hitkishorei, O. costatum, O. gujaratense* and *O. parvifolium* has been provided in Table 1. Besides the shape of rhizome, acropetal and basipetal pattern of root emergence on the rhizome of *Ophioglossum* species has been recognized as feature of taxonomic importance and this character was used to distinguish different species of this fern^31,35^. *O. trilokinathii* sp. nov. shares the character of basipetal pattern of emergence of young roots with *O. costatum* but later species is larger in size with subglobose rhizome whose bottom part being devoid of roots in contrast to the former one which is smaller in size with subglobose-tuberous rhizome.

In majority of taxa, the mesophyll tissue is homogenous made up of isodiametric spongy cells. The trophophylls in the present taxon are in rosette and lie flat on the substratum resulting into the unequal illumination of the two surfaces of the blades which lead the differentiation of mesophyll tissue into palisade and spongy parenchyma. Such differentiation of mesophyll is unknown in species of *Ophioglossum* except *O. nudicaule*^36^. The structure and design of mesophyll tissue in the plant leaf is one of the key traits playing important role in the regulation of photosynthesis. Elongated chlorenchyma cells of palisade layer with large amount of chloroplast which tend to stay very close to the walls of the cells to harvest maximum amount of illumination enable the plants to make optimal advantage of available light. Palisade parenchyma also helps in distribution of light more uniformly to chloroplast within the cell^37^.

Owing to significant variation among species in stomatal features particularly the stomatal shape, shape and ornamentation of inner margin of outer stomatal ledge, they have been used taxonomically by the earlier workers^38^ in separating the complex taxa of Dryopteridaceae in pteridophyta. Epidermal features and mesophyll characteristics have also been suggested by various researchers as supporting evidences in differentiating some species of genus *Ophioglossum*^17,22^. Outer stomatal ledges prevent wide opening of the stomatal pore and its lifting above leaf epidermis^39^. This lip like structure around the stomatal pore also helps in preventing water loss by sealing the pore during physiological stress^40,41^. Thus, the differentiation of mesophyll tissue and stomatal ledges are the features which provide survival potential to the new taxon as it is a heliophyte occurring in open terrestrial habitat.

Sporoderm features were treated as of taxonomic importance in distinguishing the European species of *Ophioglossum*^42,43^. Spore coat structure could be valid mark for identification of some *Ophioglossum* species^43^. Spore characters are not easily prone to environmental influences compared to vegetative features and are thus more dependable in systematic consideration^44^. Some workers^45^ have concluded for Japanese species of *Ophioglossum* that the ornamentation pattern of a particular species collected from different localities and with distinct morphological variability possesses a specific pattern of spore exine.

Spore morphology has now assumed a significant role in pteridophytic taxonomy. Several workers^9,23,34,46–51^ have treated the exine ornamentation pattern as the most reliable character for systematic purposes in *Ophioglossum.* Thus, spore studies fully justify the relevance and validity of SEM investigation in the species delimitation of *Ophioglossum*. Three types of exine ornamentation patterns namely reticulate, verrucate and scabrate are known in species of *Ophioglossum*^52^. The exine ornamentation pattern recorded in *O. trilokinathii* sp. nov. is entirely different consisting of thick and raised muri enclosing large hexagonal or irregular areas on the proximal and distal poles of the spore. Such an exine ornamentation is not known so far in any of the species of the genus *Ophioglossum.*

DNA barcoding has been used increasingly for shedding light on species delineation in many fern species including *Ophioglossum.* For a valid plant barcode, it is emphasized to include conservative coding region like *rbcL* together with rapidly evolving non-coding region like *trnL-F*^53^. Similarly, The *psbA-trnH* intergenic region is among the most variable regions in the angiosperm chloroplast genome and is a popular tool for plant population genetics and species level phylogenetics. This region has also been suggested to be suitable for DNA barcoding studies^54^. Various researchers have successfully used these DNA barcodes for molecular identification of *Ophioglossum*^20–23^.

In the present study, the comparative phylogenetic analysis of seemingly allied species of *Ophioglossum* suggests considerable degree of differences between *O. trilokinathii* and *O. hitkishorei* with respect to *rbcL* and *psbA-trnH* regions as confirmed by determination of evolutionary divergence (p-distance) and percent identity matrix. The sequence difference between *O. trilokinathii* and *O. hitkishorei* in terms of percent identity matrix was as high as 6.9% for *rbcL* region. The sequence data of *O. sp. SAD 2020a* were available in GenBank only for *psbA-trnH* region and not for the remaining two regions (*rbcL* and *trnL-F*). Although, the data on p-distance could not provide evolutionary divergence between *O. trilokinathii* and *O. sp. SAD-2020a* respectively, percent identity matrix suggested considerable differences in the nucleotide sequences of these specimens with respect to *psbA-trnH* region. Furthermore, the morphological description of *O. sp. SAD-2020a* was not available in the published literature. Based on the information available in NCBI database with respect to the sequence data of psbA-trnH marker, *O. SAD2020a* specimen belongs to Kerala (Southern India) while *O. trilokinathii* is reported from northwestern part of India (Rajasthan). Both the regions have altogether different bio-geoclimatic conditions.

Taking all the attributes including habitat, ecology, morphology, foliar anatomy, stomatal features, palynology and molecular phylogenetic data into consideration we conclude that the *Ophioglossum* specimen collected from plateau region of Rajasthan represents a hitherto undescribed species thereby warranting its establishment as *O. trilokinathii* sp. nov.

## Methods

### Morphology

Fresh plants were collected in polythene bags from Mainal (25° 5^′^ 15^′′^ N, 75° 10^′^ 01^′′^ E, elevation ~ 507 m) of Chittorgarh district, Rajasthan, India. Permission for the plant sample collection was obtained from the Forest Department, Chittorgarh. Photographs of plants growing in nature were taken. Field observations such as habitat, elevation, latitude and longitudes were recorded. Material was washed with water and fixed in Formalin, Acetic acid, Alcohol (FAA, 5:5:90) solution for further examination in laboratory. Herbarium specimens were prepared following standard method of drying, pressing and mounting on sheets. The specimens were deposited in the Herbarium, Mewar University, Chittorgarh, Rajasthan (voucher ID number B. L. Yadav, C. B. Gena & Kanta Meena 65 MUCR 00036). Morphological observations were based on fresh as well as herbarium material. All the measurements were recorded in metric scale. Comparison with other allied species of *Ophioglossum* collected from Rajasthan, India and with the digital images available online from LINN and Kew Herbarium Catalogues^55,56^ was made following published literature and expert opinion. The morphological features of spores were studied without acetolysis as this technique has not been recommended in family Ophioglossaceae because it dissolves the fine structures of the exine^48^. Formal identification of plant specimens was carried out by the authors (B. L. Yadav and C. B. Gena) which was further verified and confirmed by Professor T. N. Bhardwaja, Pteridologist & Former Vice-Chancellor, V. M. Open University, Kota, Rajasthan, India. All the investigations pertaining to the plant species description were performed in accordance with relevant guidelines and regulations.

### Scanning electron microscopic observations

Spore as well as leaf samples from mature plants were used for Scanning Electron Microscopic (SEM) analysis. Spores were mounted on aluminium stubs with double-adhesive tape and were coated with thin layer (200–400 dA) of gold–palladium using Sputter Coater model Quorum-Q-150 TES. The prepared samples were then examined using the SEM (Make FEI; Model Nova NanoSEM-450) in the SEM laboratory of Material Research Centre, Malviya National Institute of Technology, Jaipur, Rajasthan. For SEM images of stomata, samples of leaves (small piece of leaf) were taken in place of spores.

### Venation pattern

The entire leaves were cleared in 10% sodium hydroxide (NaOH) solution for 24 h following which they were repeatedly washed and transferred to a supersaturated solution of Chloral hydrate for 24 h until rendered transparent﻿^57^. After thorough washing in water, leaves were stained in aqueous safranin and mounted in 50% glycerin. Venation pattern was observed from glycerin mounted leaf under light microscope (Magnus Optosystems India).

### Foliar anatomy

Mature leaves (trophophylls) from the FAA fixed plants were taken. Hand cut sections were stained in 1% aqueous safranin before mounting on slides in 50% glycerine and then observed under light microscope. Photomicrographs of these temporary preparations were taken using camera Model E-PL 1 (Olympus).

### Molecular characterization

#### Genomic DNA extraction, PCR amplification and sequencing

The genomic DNA from the sterilized plant material was extracted using the method suggested by Doyle and Doyle﻿^58^. Extracted DNA was quantified employing the method of Sambrook et al﻿*.*^59^. Purity of the DNA was further determined by electrophoresis in agarose gel (0.8%). PCR amplification of three chloroplast DNA (cpDNA) markers (*trnL-F*, *rbcL* and *psbA-trnH*) was carried out using the extracted DNA as template. A single PCR protocol was recruited in respect of all three chloroplast regions. In the process, 20 μL reaction mixture containing extracted genomic DNA template (2 μL) (1:10 dilution of the extracted DNA), forward primer (1 μL), reverse primer (1 μL), 1 × final concentration of ReadyMix Taq PCR reaction mix (SIGMA-ALDRICH) (10 μL) and nuclease free water (6 μL). The PCR was carried out in Thermal cycler (BIO-RAD iCycler). PCR program settings included: 94 °C for 4 min, 30 cycles of 94 °C for 30 s, 50 °C for 30 s, and 72 °C for 1.30 min, and a final elongation step at 72 °C for 10 min. Amplified chloroplast markers (*trnL-F*, *rbcL* and *psbA-trnH*) were visualized on 1% agarose gel under UV light by staining with ethidium bromide. Amplified PCR products were purified using GenElute PCR Clean-up kit (SIGMA-ALDRICH) and sequenced at Barcode Biosciences, Bangalore.

#### Alignment and phylogenetic analysis

Newly generated sequence data were aligned using Gene Tool v1.0 and their contigs were prepared. NCBI-BLAST search was conducted to reveal the sequence identity. The Sequence data were then deposited to NCBI database (Accession numbers: *trnL-F*, MW081148; *rbcL,* MW081146; *psbA-trnH,* MW081147).

Further, nucleotide sequences of *Ophioglossum* species (28 sequences of *rbcL*, 18 sequences of *trnL-F* and 22 sequences of *psbA-trnH*) were retrieved from the GenBank and used for comparative analysis with nucleotide sequences of *O. trilokinathii* sp. nov. *Botrychium lunaria* of the same family was included in the analysis as an outgroup taxon. Nucleotide sequences of all three chloroplast markers as mentioned above were subjected to pairwise alignment and multiple sequence alignment (MSA) using Clustal-W embedded in MEGA X with default settings. Also, MEGA X was used to find out the best fit model for phylogenetic analyses^60^. The evolutionary history was inferred by using the Maximum Likelihood method wherein the models with the lowest BIC scores (Bayesian Information Criterion) were considered to describe the substitution pattern the best and selected as best-fit model^61^. Accordingly, K2 (Kimura-2 parameter) model was found to be the best-ft model in case of the *rbcL* dataset whereas T92 (Tamura-3 parameter) was the best-fit model for of *psbA-trnH* and *trnL-F* datasets. The bootstrap consensus tree inferred from 1000 replicates^62^ was taken to represent the evolutionary history of the taxa analyzed^62^. Initial tree (s) for the heuristic search were obtained automatically by applying Neighbor-Join and BioNJ algorithms to a matrix of pairwise distances estimated using the corresponding best-fit model, and then selecting the topology with superior log likelihood value. A discrete Gamma distribution was used to model evolutionary rate differences among sites (5 categories (+ *G*, parameter = 10.3173 for *psbA-trnH*, 1.8565 for *rbcL and* 3.7709 for *trnL-F* datasets). All positions with less than 95% site coverage were eliminated, i.e., fewer than 5% alignment gaps, missing data, and ambiguous bases were allowed at any position (partial deletion option). The trees with the highest log likelihood (−2272.44 for *psbA-trnH*, −2007.65 for *rbcL and* −1758.23 for *trnL-F* datasets) are shown as Figs. 5, 6 and 7. The percentage of replicate trees in which the associated taxa clustered together in the bootstrap test (1000 replicates) are shown next to the branches in the trees^61^. The evolutionary divergence (p-distance) between sequences was calculated using MEGA X^60^. The rate variation among sites was modeled with a gamma distribution (shape parameter = 1). Codon positions taken into consideration were 1st + 2nd + 3rd + Noncoding. All positions with less than 95% site coverage were removed with partial deletion option. In total, there were 453, 226 and 297 positions in the final datasets corresponding to *rbc*L, *trnL-F* and *psbA-trnH* regions respectively. The percent identity matrix was calculated using Clustal Omega (https://www.ebi.ac.uk).

## Supplementary Information

Supplementary Figure S1.

Supplementary Tables.

## Data Availability

All the generated sequences have been submitted to the GenBank with accession number as *trnL-F*, MW081148; *rbcL,* MW081146; *psbA-trnH,* MW081147.
