# Supplementary material for: Discovery of a new species of Adder’s tongue fern from India with comparative analysis of morphological and molecular attributes
Source: Sci Rep. 2021 Dec 22;11:24396. doi: 10.1038/s41598-021-03231-w (PMC8695580; doi:10.1038/s41598-021-03231-w)
Supplement: Supplementary file 1 — Supplementary Figure S1. [file 41598_2021_3231_MOESM1_ESM.docx]

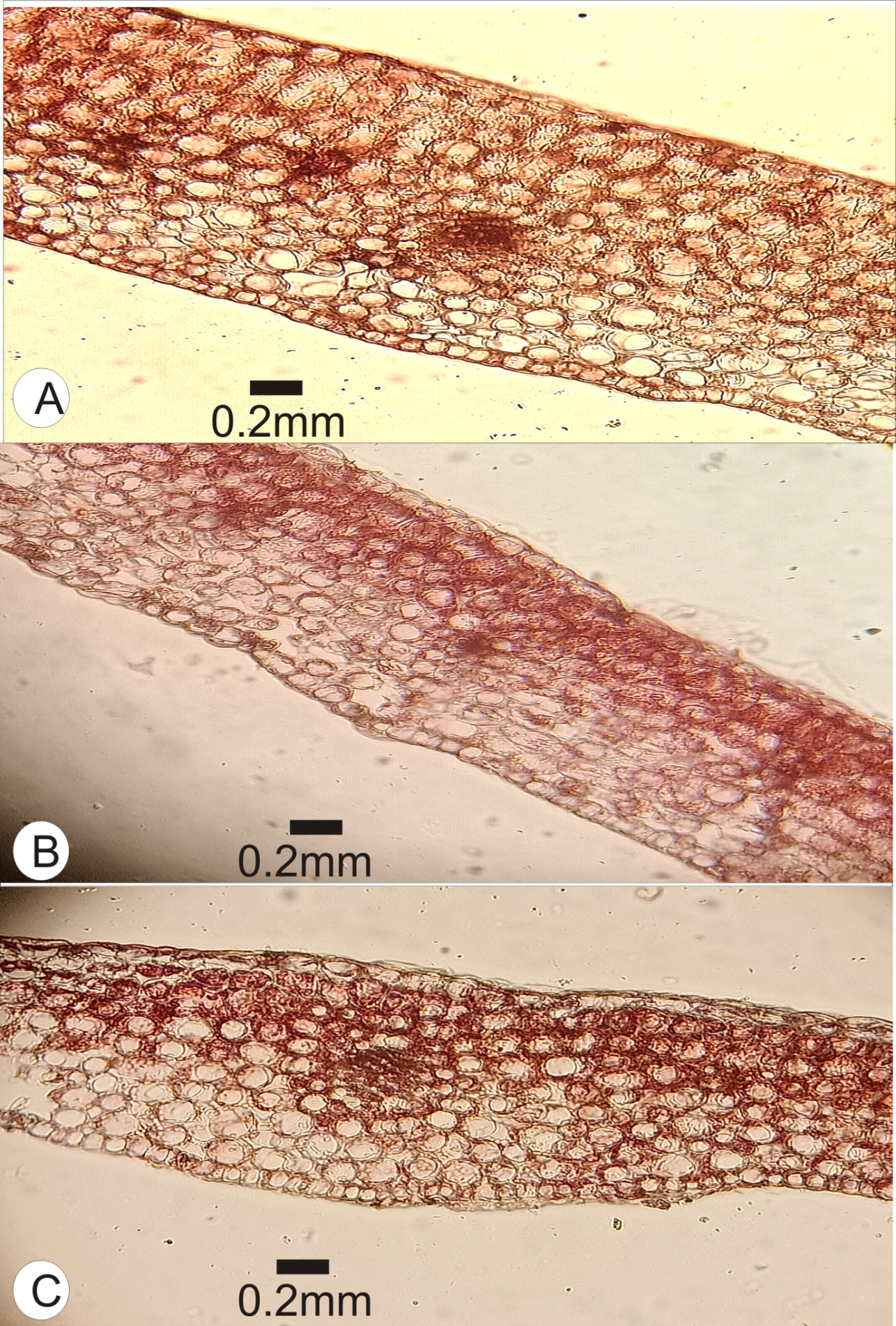


**Supplementary Figure S1**. Leaf (Trophophyll) anatomical features of three species of *Ophioglossum* (A). *O. trilokinathii* sp. nov. (B). *O. costatum* (C). *O. gujaratense.*
